# High Grazing Pressure of Geese Threatens Conservation and Restoration of Reed Belts

**DOI:** 10.3389/fpls.2018.01649

**Published:** 2018-11-12

**Authors:** Elisabeth S. Bakker, Ciska G. F. Veen, Gerard J. N. Ter Heerdt, Naomi Huig, Judith M. Sarneel

**Affiliations:** ^1^Department of Aquatic Ecology, Netherlands Institute of Ecology, Wageningen, Netherlands; ^2^Department of Terrestrial Ecology, Netherlands Institute of Ecology, Wageningen, Netherlands; ^3^Waternet (Water Board Amstel Gooi en Vecht), Amsterdam, Netherlands; ^4^Department of Ecology and Environmental Sciences, Umeå University, Umeå, Sweden; ^5^Ecology and Biodiversity Group, Utrecht University, Utrecht, Netherlands; ^6^Plant Ecophysiology Group, Utrecht University, Utrecht, Netherlands

**Keywords:** *Anser anser*, aquatic plant, exclosure, herbivory, landscape configuration, *Phragmites australis*, restoration, wetland

## Abstract

Reed (*Phragmites australis* (Cav.) Trin. ex Steud.) beds are important habitat for marsh birds, but are declining throughout Europe. Increasing numbers of the native marsh bird, the Greylag goose (*Anser anser* L.), are hypothesized to cause reed bed decline and inhibit restoration of reed beds, but data are largely lacking. In this study, we experimentally tested the effect of grazing by Greylag geese on the growth and expansion of reed growing in belts along lake shorelines. After 5 years of protecting reed from grazing with exclosures, reed stems were over 4-fold denser and taller than in the grazed plots. Grazing pressure was intense with 50–100% of the stems being grazed among years in the control plots open to grazing. After 5 years of protection we opened half of the exclosures and the geese immediately grazed almost 100% of the reed stems. Whereas this did not affect the reed stem density, the stem height was strongly reduced and similar to permanently grazed reed. The next year geese were actively chased away by management from mid-March to mid-June, which changed the maximum amount of geese from over 2300 to less than 50. As a result, reed stem density and height increased and the reed belt had recovered over the full 6 m length of the experimental plots. Lastly, we introduced reed plants in an adjacent lake where no reed was growing and geese did visit this area. After two years, the density of the planted reed was six to nine-fold higher and significantly taller in exclosures compared to control plots where geese had access to the reed plants. We conclude that there is a conservation dilemma regarding how to preserve and restore reed belts in the presence of high densities of Greylag geese as conservation of both reed belts and high goose numbers seems infeasible. We suggest that there are three possible solutions for this dilemma: (1) effects of the geese can be mediated by goose population management, (2) the robustness of the reed marshes can be increased, and (3) at the landscape level, spatial planning can be used to configure landscapes with large reed bed reserves surrounded by unmown, unfertilized meadows.

## Introduction

Riparian zones located at the interface between aquatic and terrestrial ecosystems, are generally rich in biodiversity (Nilsson and Svedmark, 2002; Brauns et al., 2007; Valkama et al., 2008). However, reed (*Phragmites australis* (Cav.) Trin. ex Steud.) beds throughout Europe have been reported to degrade and decline in size (Ostendorp et al., 1995; van der Putten, 1997; Vermaat et al., 2016). Reed beds are important habitat for marsh birds, of which many species are a conservation concern (Graveland, 1998; Vermaat et al., 2008; Beemster et al., 2010; Voslamber and Vulink, 2010). Therefore, it is of utmost importance that existing reed beds are being protected and deteriorated reed beds are being restored. However, recently, a new conservation dilemma emerged with regard to reed bed protection and restoration. The recent increase in the number of Greylag geese (*Anser anser* L.) causes concern about their impact on reed vegetation during summer (Vermaat et al., 2016; Buij et al., 2017).

In the past, the degradation and decline of reed beds has been mainly attributed to adverse abiotic conditions for reed growth, including eutrophic water and sediment, toxicity of accumulated dead plant material and a lack of favorable water level dynamics (Ostendorp et al., 1995; van der Putten, 1997; Lamers et al., 2015). Reed is a clonal plant, which can be long-lived, reed regenerates by germination on moist sediment, appearing just above the water table (Packer et al., 2017). However, other goose species have been shown to cause strong and negative impacts on wetland vegetation in arctic tundra, including Lesser snow geese (*Chen caerulescens caerulescens* L.) in North-America (Jefferies et al., 2004) and Pink-footed geese (*Anser brachyrhynchus* Baillon) in the European high arctic (Speed et al., 2009). In analogy, we hypothesize that Greylag geese may have similar negative impacts on reed beds in temperate wetlands, which may jeopardize the conservation and restoration of reed beds.

Traditionally, Greylag geese were mainly found in western and southern Europe in autumn and winter, where Greylag geese foraged on belowground parts of several helophyte species, including *Scirpus maritimus* L. (Amat, 1995; Esselink et al., 1997) and *Spartina anglica* C.E.Hubb (Bakker et al., 1999). However, over the last decades Greylag geese have increasingly bred in western Europe, creating large resident summer populations (Klok et al., 2010). In The Netherlands, numbers of resident breeding and molting summer geese have increased from about 50,000 in the late 1990's to over 200,000 since 2010 (SOVON, https://www.sovon.nl/nl/soort/1610). The increase in the amount of summer geese in The Netherlands has been attributed to the success of the Oostvaardersplassen wetland as a molting and breeding site (Loonen et al., 1991) which has served as a source for the breeding population in The Netherlands. However, since then the change in land-use where increasing fertilization of agricultural meadows yield a very high quality food for geese is fueling the population growth of summer geese (Van Eerden et al., 2005). Whereas the geese prefer reed beds as breeding and molting sites, they do not strictly depend on them. The higher abundance of geese in summer can result in high grazing pressure on the aboveground parts of helophytes, including reed. Grazing pressure on reed is especially high during the molting period in May and June (Dingemans et al., 2011), as the geese then preferably stay on open water to avoid predation by terrestrial predators (Fox and Kahlert, 2000). This coincides with the timing of emergence of fresh shoots of reed (Loonen et al., 1991). By consuming the reed parts that emerge above the water, the geese would destroy potential breeding habitat of marsh bird species that depend on this type of reed, including the Great reed warbler (*Acrocephalus arundinaceus* L.) and the Purple heron (*Ardea purpurea* L.), target species for conservation management (Graveland, 1998; Prokesova and Kocian, 2004). Geese graze reed stems above the water level in particular while swimming on the water when they are molting. Greylag geese can graze reed stems when walking on shore, but need open water to drink frequently, as reed is very hard to consume, in part due to the toughness of the stems and leaves, which contain high levels of silica (Van Eerden, 1997).

Therefore, nature managers are facing a dilemma: should they reduce numbers of native Greylag geese to conserve reed beds that are needed as foraging and breeding habitat for other endangered marsh birds?

In this study, we tested the effect of grazing by Greylag geese on the growth and expansion of reed growing in belts along lake shorelines. We hypothesized that (1) expansion of existent reed belts is inhibited by the presence of Greylag geese, (2) temporal fencing would make the reed belts resistant to grazing, as temporal exclusion would allow the reed stands to increase their belowground resources, which could increase their capacity for re-growth after grazing (c.f. Smit et al., 2010) and the tall and dense reed stands that develop when protected from grazing may be less attractive for the geese to graze upon, as they preferably graze on the edge of stands of their food plants (Bakker et al., 1999) (3) geese removal by management stimulates reed growth and (4) restoration of a reed stand in a site where reed is absent in the presence of geese could only be achieved when introduced reed plants are protected from grazing. Summarizing, we hypothesized that Greylag geese would strongly inhibit reed growth, establishment and expansion and thus removal of geese by fencing or management would improve reed growth, establishment and expansion. We experimentally excluded Greylag geese from existing and introduced reed vegetation in two freshwater lakes in the center of The Netherlands and measured their impact on the number of reed stems, stem height and the expansion of reed into open water. We subsequently removed part of the fences to test robustness of the reed stand to withstand grazing and tested whether removal of geese by active chasing management improved reed growth.

## Materials and methods

### Study area

The study was conducted in the Loenderveen area in the center of The Netherlands, in two adjacent lakes: Lake Terra Nova (52°13′07″N, 5°02′27″E) and Lake Waterleidingplas (52°12′55″N, 5°02′33″E). Both lakes are owned and managed by Waternet, the drinking water company for Amsterdam and surroundings. Lake Terra Nova (85 ha) is a shallow eutrophic peat lake, while Lake Waterleidingplas (160 ha) is a shallow mesotrophic lake with a sand bottom. Lake Waterleidingplas is used as a drinking water reservoir and has good water quality, whereas Lake Terra Nova is used for water storage and has undergone intensive restoration management to improve its water quality and biodiversity (Ter Heerdt and Hootsmans, 2007; Immers et al., 2015).

The banks of Lake Terra Nova once were fringed with helophyte belts. Around the year 2000 these had disappeared due to a combination of shade cast by trees, erosion, turbid water and, presumably, herbivorous bird grazing. In 2006, to encourage the expansion of helophytes and decrease bank erosion, many banks were protected with a wooden wall, about 2 m in front of the bank, protruding 10–20 cm above the water at average water level (Supplementary Figure 1A). To improve light conditions for the helophytes in the shallow, clear and tranquil water behind the bank protection, all large trees were cut from the islands. This also prevented bank erosion by tree collapse. At the start of the study Lake Waterleidingplas had large scale stands of mainly reed and a few other helophyte species, which grow partly on the shore and extend up to about 2 m away from the shore into the open water (Supplementary Figure 2A).

The Loenderveen area is an important fresh water lake system for water birds, as it is a large natural area which is closed to the public. The reed belts offer a suitable breeding habitat for many marsh bird species, including the Greylag goose and the Great reed warbler (*Acrocephalus arundinaceus*) (unpubl. data G. N. J. Ter Heerdt). The Purple heron (*Ardea purpurea*) breeds in the reed belt in a third adjacent lake, Lake Loenderveen Oost, which was not included in this study. The latter two bird species are threatened in The Netherlands and the Loenderveen area is one of the hot spots for these birds in the country. Therefore, its management is aimed at protecting, and preferably increasing, the reed belts in this area.

Lake Terra Nova hosts a breeding population of Greylag geese that nests on the small islands in the lake, estimated to include approximately 150 pairs in 2005, which increased to about 200 in 2013 (unpubl. data G.J.N. Ter Heerdt). Systematic counts of geese on Lake Terra Nova were not available. The amount of Greylag geese on Lake Waterleidingplas were counted through the reed growing season from May through August on a monthly or biweekly basis by experienced bird watchers in 2006–2013, with the exception of 2008 and 2011, when no data were available (see Table 1 for maximum numbers of Greylag geese counted). Both study lakes are visited by two exotic goose species, which occur in much lower numbers, Canada goose (*Branta candensis* L.) and Egyptian goose (*Alopochen aegyptiaca* L.). Muskrats (*Ondatra zibethicus* L.) occur in the area and are trapped. Based on catches, the biomass density of muskrats for adjacent Lake Loenderveen Oost was estimated to be on average 0.11 kg/ha in 2011 and 2012 (Sarneel et al., 2014a), whereas data for the two study lakes were not available.

**Table 1:** Maximum number of Greylag geese counted during monthly or bi-weekly counts in Lake Waterleidingplas during the study period.

	**2006**	**2007**	**2009**	**2010**	**2012**	**2013**
May	n.a.	510	425	360	1,309	34
June	390	885	797	697	2,361	77
July	450	36	105	480	234	450
August	120	723	13	241	123	34

*No data were available from 2008 and 2011 and from May 2006. N.a., not available*.

### Experimental design

We performed three consecutive experiments in Lake Waterleidingplas to test the effect of Greylag geese presence on existing reed belts. We tested the effect of Greylag geese on reed establishment when reed is absent by experimentally introducing reed plants in adjacent Lake Terra Nova.

#### Geese exclusion from existing reed belts

**Figure 1:**
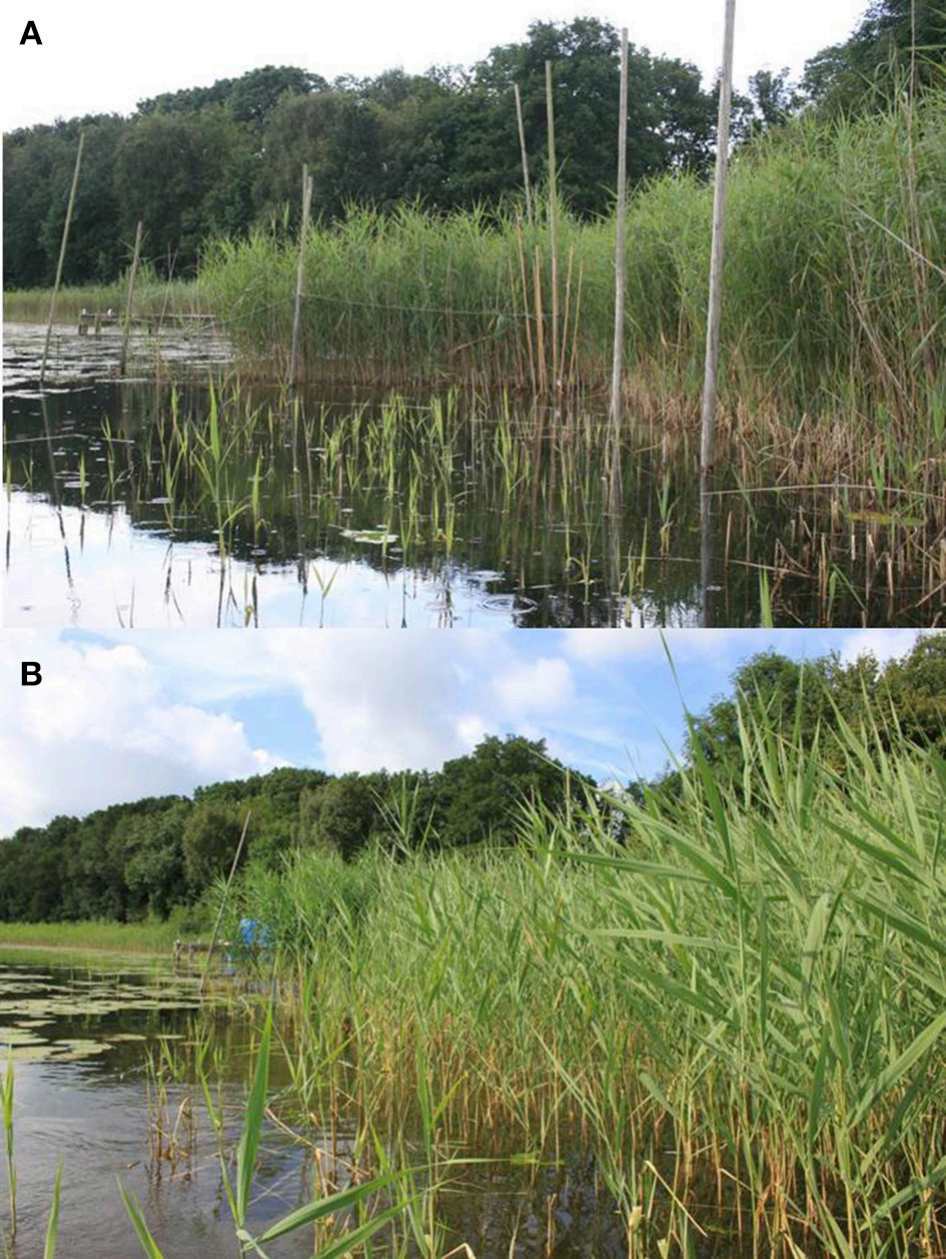
Exclosure design in the study area, Lake Waterleidingplas. **(A)** An exclosure is visible in the background, marked by 6 poles, with the horizontal upper line of the net just visible across the poles. The exclosure runs from the reed belt on the right into the open water to the left. A tall reed stand is visible inside the exclosure. In front of the exclosure, the adjacent control plot is visible, the two poles on the right mark the shore side of the control plot, from there the plot runs to the left, toward the open water. Sparse and short reed stems are visible in the control plot, which have been grazed in spring and early summer and are now resprouting, 5 August 2008. **(B)** The same exclosure as in **(A)**, from the same perspective, but now 5 years later on 31 July 2013, after geese have been actively chased away in spring and early summer by the local water board authority. The exclosure is now hardly visible, as the reed in the control plot and surrounding area has grown tall in the absence of goose grazing.

**Figure 2:**
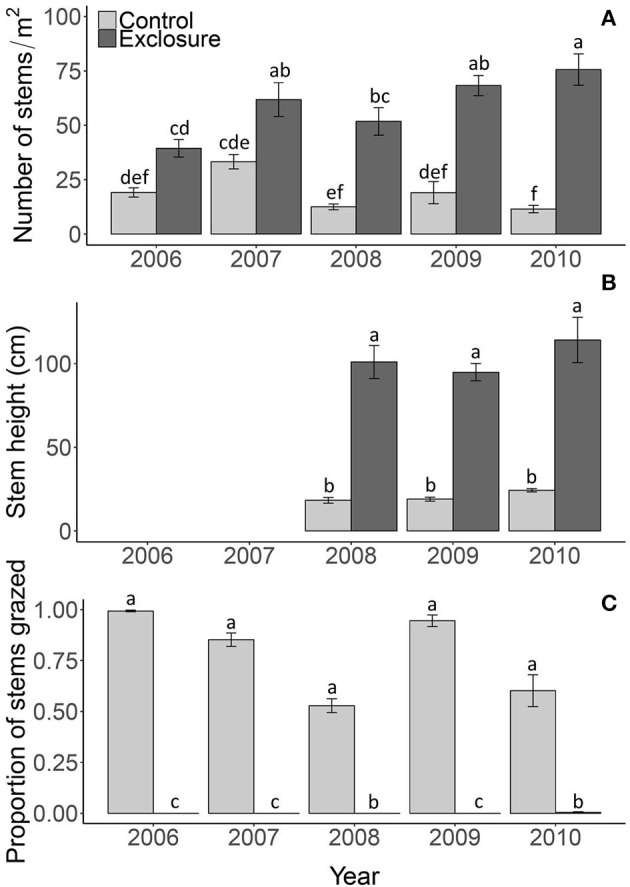
Reed density **(A)**, stem height above the water **(B)** and proportion of stems grazed **(C)** in the control plots and exclosures. Data are means ± SE, *n* = 6. Different letters indicate significant differences among the control and exclosure treatments and years at *P* < 0.05.

**Figure 3:**
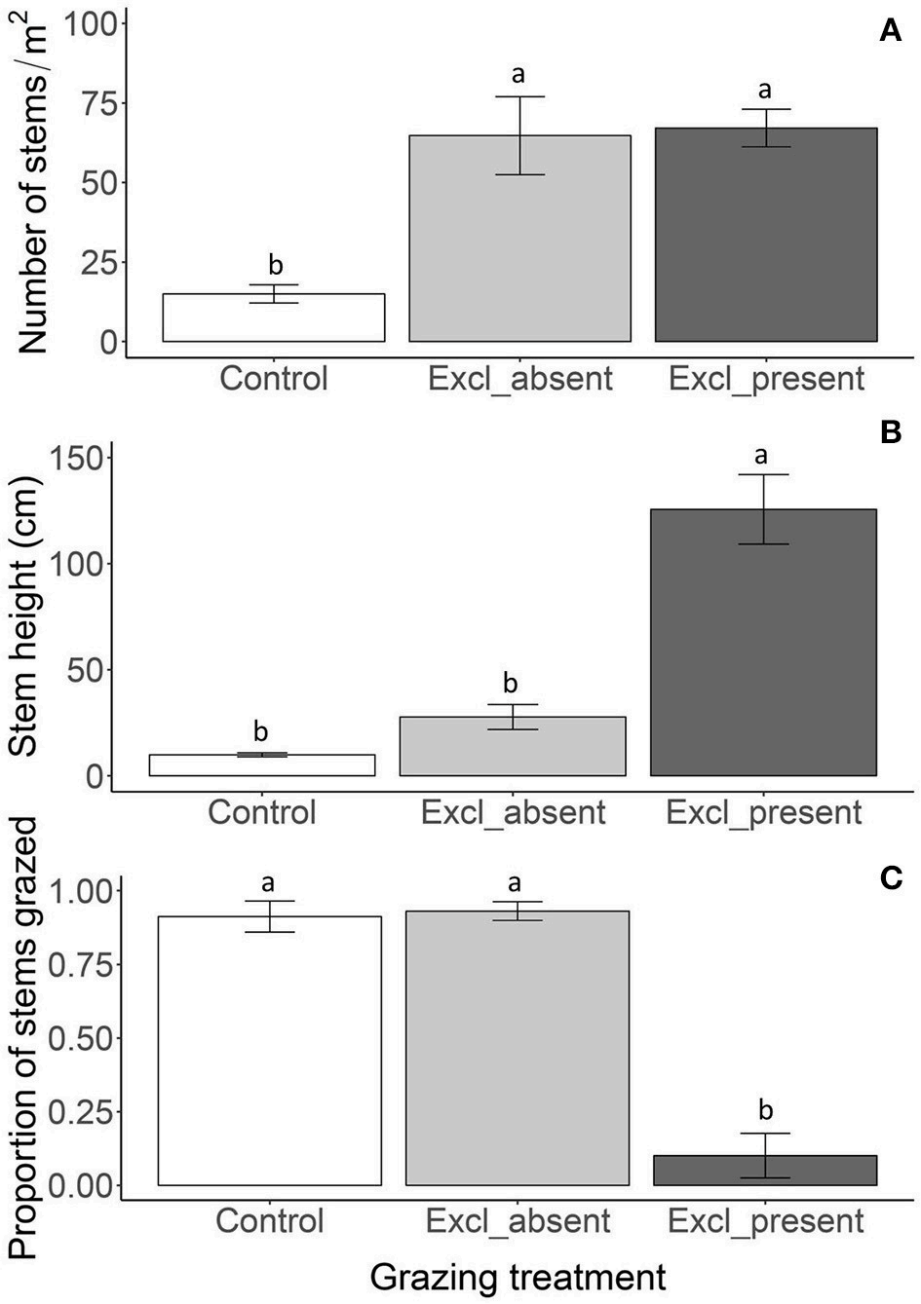
Reed density **(A)**, stem height above the water **(B)** and proportion of grazed stems **(C)** in July 2011 in the control plots, plots where exclosures were removed (exclosure_absent) and exclosures (exclosure_present). The exclosure absent treatment consisted of reed which had been protected for 5 years by fencing, whereas now fencing had been removed before the start of the growing season. The exclosure present treatment had kept its fencing. Data are means ± SE, *n* = 6 for the control plots and *n* = 3 for the exclosure present and absent treatments respectively. Different letters indicate significant differences among the grazing treatments at *P* < 0.05.

Six exclosures were built in Lake Waterleidingplas in early March 2006. Each exclosure consisted of 6 wooden poles standing in the lake at the edge of the reed belt (about 1–1.5 meter from the shore), forming a rectangle of 2 meters wide and 6 meters into the water from the shore (Figure 1). Netting (mesh size: 2.5 cm) kept Greylag geese from getting in and protected the vegetation against grazing. In addition to Greylag geese, the nets also prevented other water birds to enter the exclosures, in particular Eurasian coots (*Fulica atra* L.) and mute swans (*Cygnus olor* Gmelin), which were less numerous than Greylag geese during the study. Nets appeared at least 1 m above the water level and extended under water down to the sediment. The exclosures were covered by wire on top to prevent geese from landing in the exclosure. No geese were observed to enter an exclosure. Sporadic grazing tracks were observed on reed inside the exclosure, on the reed on the edge of the transects, closest to the nets, which was negligible in 2006–2010 (Figure 2C) and less than 10% of the stems in 2011 (Figure 3C). Next to each exclosure, a similar shaped control plot was built, consisting only of the poles (Supplementary Figure 2B), which was randomly assigned to the left or right of the paired exclosure. The distance between an exclosure and its paired control plot was about 2–5 m. The distance between pairs of exclosures and control plots was about 50 m. During the study water depth ranged from a minimum of 16 cm in the shallowest part at the shore side to a maximum of 140 cm in the deepest part at the open water side of the exclosures and control plots. Water levels were maintained at a more or less stable level, the plots never fell dry during the study.

In each exclosure and control plot we marked a transect running through the middle of the plot that consisted of 5 subplots of 0.5 m long and 0.7 m wide, starting 0.25 cm from the shore side of the plot and separated by 0.5 m from each other. In each subplot we counted the number of living reed stems, which were all green, firm stems. Last year's stems were easily recognizable as being grayish and very brittle and were not counted (Supplementary Figure 2B). We only counted the stems that appeared above the water surface. We recorded all stems of emergent helophytes in these subplots, which were *Phragmites australis, Typha angustifolia* L., and *Scirpus lacustris* L. However, more than 99% of all counted stems were *Phragmites*, hence the other species were discarded for further analyses. We recorded the stem density in the summers of 2006–2010 (between late June and early August). For each subplot we measured the height of the stem above the water in 2008–2010. Stem height was measured for three randomly selected stems in each subplot, or less if less than three stems were available in a subplot. For all measurements we separately noted whether stems were grazed. We defined stems to be grazed when leaves on the stem were grazed or when the whole stem was grazed. Greylag goose grazing leaves a fringe on the stem edge (Supplementary Figure 2B), whereas muskrats, which are also present in the area, leave a characteristic cut mark, with the “remaining stem fragment cut in a characteristic, oblique way c. 5–20 cm above the water” (Vermaat et al., 2016, p. 4). We are confident that we did not observe stems with these muskrat grazing marks in our study area.

#### Robustness of reed belts to renewed geese access

In 2011, half of the exclosures were removed in late winter, yielding three intact exclosures and three former exclosures without protective nets after 5 years of fencing. We compared the parameters of the reed stands in the control plots with those in the three intact exclosures and the three exclosures from which the fences were removed.

#### Chasing of geese to test recovery potential of reed

In 2013, the local water authority Waternet started a program to chase away geese from the lake. Two men were boating across the lake during day light, actively chasing away geese. The chasing management was done from mid-March to mid-June. This reduced the number of molting geese from a maximum observed in 2012 of more than 2300 geese to less than 50 during the active chasing period in 2013 (Table 1). Furthermore, in 2013, all eggs and nests were removed by the water authority, after which most adult geese left the colony. We measured the response of the unprotected reed in the control plots to the removal of geese by comparing reed parameters between 2012 (no chasing management) and 2013 (with chasing management) for the control plots accessible for the geese.

#### Planting reed

To test whether a reed belt could be restored where it had been absent, we introduced reed plants in the shorelines of Lake Terra Nova on banks with additional bank erosion protection as here growing conditions for reed would be most suitable. We planted reed at five locations on three islands. At each location, we planted seven commercially obtained seedlings of approximately 25 cm tall along 1 m bank in June 2006 (Supplementary Figure 1B). We placed a 2 × 2 m exclosure around these seedlings (consisting of nets with mesh size 2.5 cm attached to four poles), which was half on the bank and half in the water (Supplementary Figure 1C). The exclosures protruded at least 75 cm above the water and the heavy linings of the nets would keep the bottom under water at the sediment. We planted another seven seedlings next to each exclosure (at 1–2 m distance) in a paired control plot accessible to geese, and marked this plot with one pole in the left corner on the bank.

We followed the development of the seedlings by counting the number of living reed stems per square meter on the bank after planting, and at the end of the growing season (late August and early September) in 2006–2008. We further measured stem length of the five tallest stems. The expansion of reed into the water was also quantified and expressed as the distance between the bank and the furthest shoot that protruded in the water.

### Data analysis

#### Geese exclusion from existing reed belts

Data from the 5 different subplots within a plot were averaged before data analyses, as separate tests including the position of the subplots indicated that the effect of subplot position was limited and gave qualitatively similar results of the impact of geese compared to models where data from subplots were averaged. To test the effect of subplot position, we used general linear models with grazing treatment (exclosure vs. control), distance to the shore and sampling year as fixed factors. Distance to the shore often had a main effect on the parameters measured, with more grazing damage, lower stems and less stems further away from the shore. Yet, there were generally no significant (*p* < 0.05) interactions between distance to the shore and any of the other fixed effects indicating that the magnitude and direction of the grazing impact was the same at all distances. Only for the proportion of grazed stems the impact was stronger with increasing distance from the shore. Since in our work we were not interested in distance *per se*, we averaged the data from the 5 different subplots within a plot before data analyses for simplicity. We used general linear models to test the impact of grazing on stem density, stem height and proportion of stems grazed. Grazing, year and their interaction were used as predictor variables and plot as random factor. We used pairwise least square means comparisons to test how the interaction between grazing treatment and year affected the response variables.

#### Robustness of reed belts to renewed geese access

To test the robustness of reed protection we used a general linear mixed model with grazing treatment (3 levels: control, exclosure present, exclosure removed) as a predictor variable and plot as a random factor. Response variables were stem density, stem height and proportion of stems grazed in 2011, i.e., the year in which half of the exclosures were removed before the growing season started. We used pairwise least square means comparisons to test which grazing treatments were significantly different from each other.

#### Chasing geese to test recovery potential of reed

To test the impact of geese removal on reed recovery we used data from the 6 control plots (i.e., plots that had been grazed throughout the experimental period) from 2012 (geese were not chased) and from 2013 (geese were actively chased). We used a general linear model with year as a predictor variable and stem density, stem height and proportion of stems grazed as response variables.

#### Planting reed

To test the development of the planted reed in Lake Terra Nova, stem density and stem height were analyzed using a repeated measures ANOVA. Grazing treatment and year were added as fixed, within subject factors, and plot location was added as subject on which the measurements were repeated over the years. Because in the control plots there were very few stems which colonized the water, the colonization distance was not further analyzed and only graphically represented. When an interaction was found, we performed a Tukey HSD *post-hoc* test (on an ANOVA containing treatment, time and location and an interaction between treatment and time as fixed effects).

Before all analyses, the normal distribution of residuals was checked with a qq-plot. Analyses were carried out in R version 3.2.5 (R Core Team, 2016) using the lmerTest package (Kuznetsova et al., 2013) for the data from the exclosure experiment in the existing reed belt and the ez package (Lawrence, 2016) for the data from the reed planting experiment. Degrees of freedom were estimated using the Satterthwaite estimation for the data from the exclosure experiment in the existing reed belt.

## Results

### Geese exclusion from existing reed belts

Excluding reed from grazing drastically altered the appearance of the reed vegetation (Figure 1A). Reed stem density was significantly higher in exclosures compared to control treatments where geese had access; after 5 years of protection from grazing, reed stems were over four-fold denser than in the grazed plots [Figure 2A; *F*_(1, 45)_ = 230.97; *P* < 0.001]. The difference between control and exclosure plots increased over time [Figure 2A; interaction between grazing treatment and year *F*_(4, 45)_ = 230.97; *P* < 0.001]. Reed stems were over four-fold taller inside the exclosures than outside in the control plots [Figure 2B; *F*_(1, 25)_ = 260.02; *P* < 0.001], which was not affected by year [*F*_(4, 25)_ = 2.10; *P* = 0.143] nor by the interaction between grazing treatment and year [*F*_(4, 25)_ = 0.619; *P* = 0.547]. The proportion of grazed stems was close to zero inside exclosures and varied between 50 and 100% of the stems being grazed in control plots, where geese had access [Figure 2C; *F*_(1, 45)_ = 1811.08; *P* < 0.001]. Grazing pressure varied among years [*F*_(4, 45)_ = 25.29; *P* < 0.001] and was affected by an interaction between grazing treatment and year [*F*_(4, 25)_ = 25.75; *P* < 0.001].

### Robustness of reed belts to renewed geese access

Reed stem density was similar between plots where exclosures were removed or where exclosures were still present and over three-fold lower in the control plots, indicating that renewed access of geese to exclosure removal plots did not reduce the number of stems within the first grazing season [Figure 3A; *F*_(2, 6)_ = 32.19; *P* < 0.001]. In contrast, stem height was reduced five-fold in the plots where exclosures were removed, resulting in a similar height as in the control plots, whereas stems were over a meter taller in plots where exclosures were still present. This shows that the geese immediately grazed down the reed vegetation upon renewed access to exclosure removal plots [Figure 3B; *F*_(2, 9)_ = 67.21; *P* < 0.001]. This was illustrated by the almost complete grazing of all the reed stems, which was not different between control plots and plots where exclosures were removed (Figure 3C), while few stems had grazing traces where exclosures were still present [*F*_(2, 9)_ = 54.76; *P* < 0.001].

### Chasing geese to test recovery potential of reed

**Figure 4:**
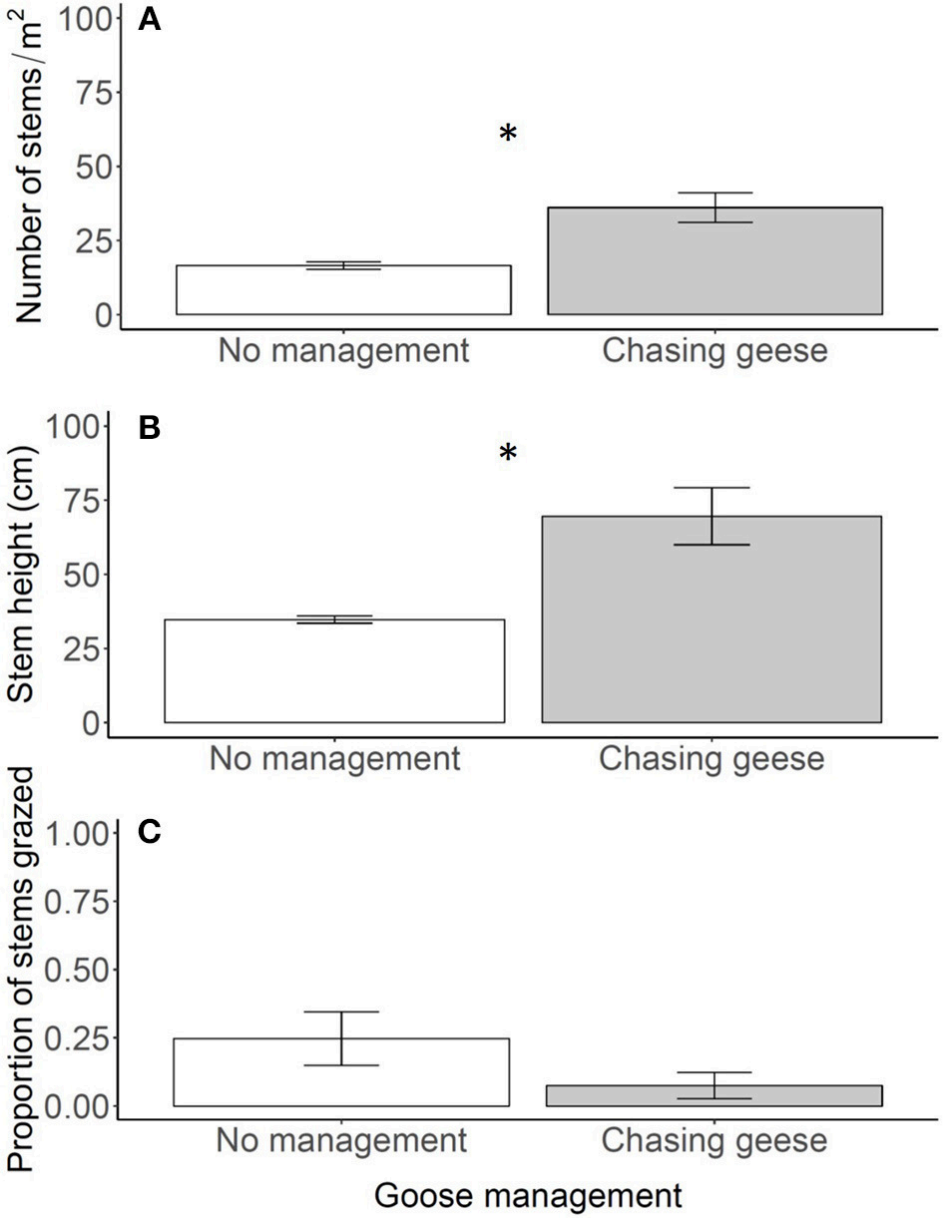
Reed density **(A)**, stem height above the water **(B)** and proportion of grazed stems **(C)** in the control plots in 2012 (no management) and 2013 (chasing geese). In 2013 geese were actively chased away, whereas they could forage undisturbed in 2012. Data are means ± SE, *n* = 6. An asterisk indicates a significant difference between years at *P* < 0.05.

Chasing away geese drastically altered the structure of the reed belt (Figure 1B). Stem density and stem height increased one- to two-fold when geese were chased away [Figures 4A,B; stem density: *F*_(1, 10)_ = 14.76; *P* = 0.003, stem height: *F*_(1, 10)_ = 12.94; *P* = 0.005]. As a result, the reed belt had recovered over the full 6 m length of the plots (Figure 1B). The proportion of grazed stems was less than 10% when geese were chased away, which was lower, but not significantly different from 2012 when there was no goose management [Figure 4C; *F*_(1, 10)_ = 2.47; *P* = 0.147], which tended to have a lower grazing pressure than the years 2006–2010 (compare with Figure 2C). No significant interactions were found.

### Planting reed

**Figure 5:**
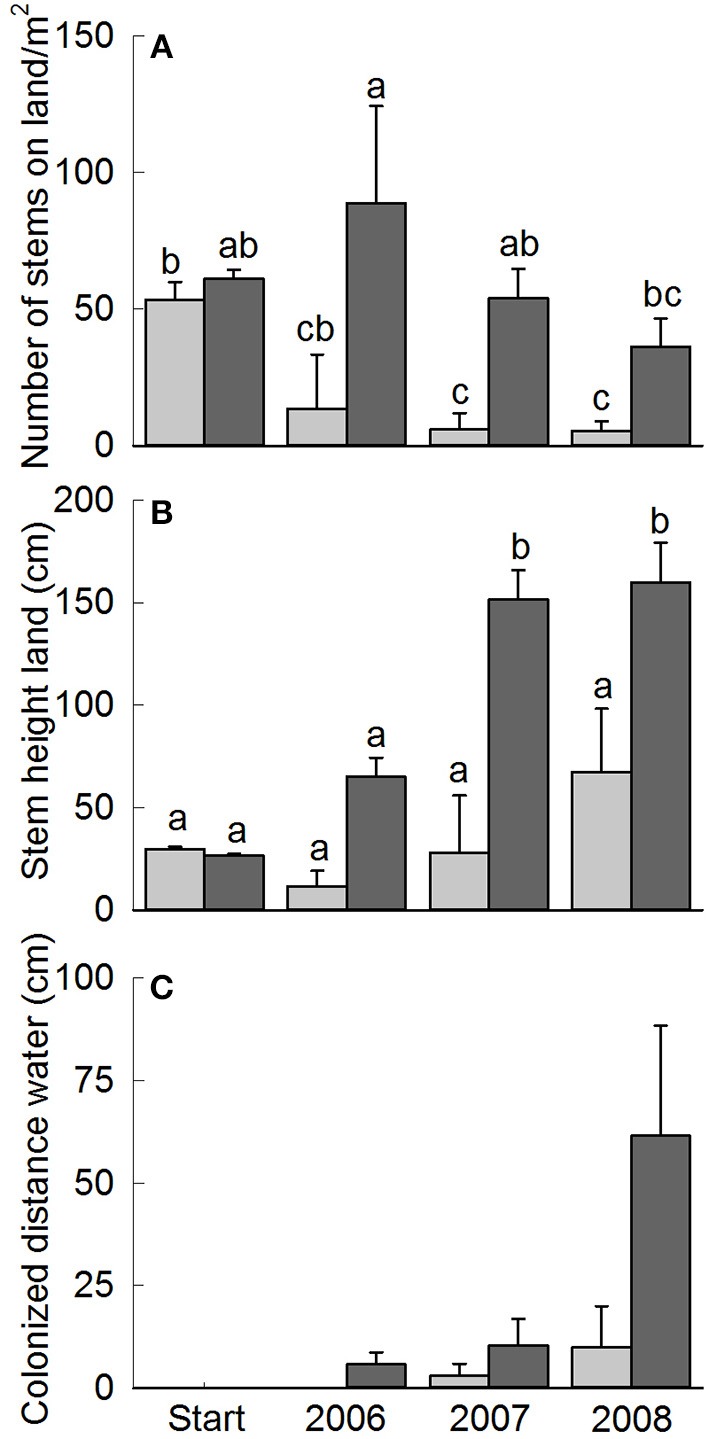
Reed density **(A)**, stem height of reed on land **(B)** and distance colonized by reed stems away from the bank into the open water **(C)** of planted reed in Lake Terra Nova. Start refers to the planting of the reed plants in June 2006, following measurements were done late August-early September. Data are means ± SE, *n* = 5. Different letters indicate significant differences among the control and exclosure treatments and years at *P* < 0.05.

At the start of the reed planting experiment, stem density did not differ between control and exclosure plots (Figure 5A). Stem density on the banks increased rapidly over the years and was higher inside the exclosures compared to controls. This increase was strongest during the first growing season, while stem density decreased a little in the following years, but it always remained six to nine-fold higher in exclosure plots compared to control plots [Figure 5A; exclosure effect: *F*_(1, 4)_ = 69.71, *P* = 0.001, year: *F*_(3, 12)_ = 4.23, *P* = 0.029, interaction between grazing treatment and year: *F*_(3, 12)_ = 5.36, *P* = 0.014]. Stem height increased over five-fold over the years to over 1.5 meter in the exclosures, whereas stem height did not change significantly in the control plots [Figure 5B; year: *F*_(3, 12)_ = 19.89, *P* < 0.001, interaction grazing treatment with year: *F*_(3, 12)_ = 7.15, *P* = 0.005]. In the last year of the study, the planted reed invaded the water in four out of the five exclosures (on average colonizing a distance of 61.5 cm ± 26.87 SE from the bank), while it invaded the water only in one of the control plots (Figure 5C). Apart from reed, species like *Eupatorium cannabinum* L.*, Alnus glutinosa* (L.) Gaertn.*, Epilobium hirsutum* L.*, Solanum dulcamara* L., and *Lycopus europaeus* L. also increased in abundance inside the exclosures (data not shown, Supplementary Figure 1D).

## Discussion

Overall, we observed a strong effect of grazing by Greylag geese on reed expansion of both existing reed stands and establishing plants. We demonstrated that removal of geese by fencing created tall, dense reed belts growing in the water along the lake shore, whereas these were largely absent in the presence of geese. However, we observed that renewed access of geese after 5 years of exclusion immediately reduced plant height and increased plant damage to levels compared to control conditions that never had been protected from grazing. Whereas the negative effects of geese on stem density take longer to establish than the effects on reed stem height, which appear immediately, temporal exclosures do not result in long-lasting reed beds beyond the immediate exclosure period. Goose chasing immediately helped to increase reed stem density and height. Hence, we confirm our hypothesis that reed growth, establishment and expansion are strongly inhibited by grazing of Greylag geese. As Greylag geese are native protected marsh birds this results in a conservation dilemma: how to conserve reed beds in the presence of high number of Greylag geese.

Recently, the role of herbivores in limiting the abundance of aquatic plants has been highlighted and quantified (Bakker et al., 2016a,b; Kollars et al., 2017; Wood et al., 2017). Aquatic herbivores remove on average 40–48% of vascular plant biomass in freshwater and marine ecosystems, which is typically 5–10 times greater than reported for terrestrial ecosystems (Bakker et al., 2016a). Our results fit in this observation that aquatic herbivores can be strong regulators of aquatic plant abundance. Whereas we did not measure reed biomass, we found an even higher reduction of reed stem density and height by Greylag geese herbivory in our study (over 75%). The finding that herbivory by geese proves to be a strong regulating factor for the abundance and establishment of reed is new. In the case of reed, its decline in Europe has been attributed in particular to a range of adverse abiotic conditions, including eutrophication and a lack of natural water level dynamics (van der Putten, 1997; Lamers et al., 2015). However, we demonstrate that the abiotic conditions are actually suitable for reed establishment in our study area, as reed grew well when protected from grazing. Therefore, we show that when abiotic bottlenecks are relieved, grazing may prevent the restoration of reed vegetation.

Aquatic herbivores may have similar impacts on the restoration and expansion of submerged freshwater vegetation and seagrass beds as we found in reed beds. High levels of herbivory by waterfowl can prevent successful restoration of submerged freshwater vegetation as the birds inhibit the colonization of plants in lakes where water quality has been restored (Bakker et al., 2013). Similarly, grazing by West-Indian manatees (*Trichechus manatus* L.) may prevent successful re-introduction of native submerged freshwater plants (Hauxwell et al., 2004). In marine ecosystems, the disappearance of large predators and the creation of marine protected reserves has locally resulted in high grazing pressure on seagrass beds by green sea turtles (*Chelonia mydas* L.) (Christianen et al., 2014). Similarly, in temperate regions, overgrazing by waterfowl may threaten the existence of seagrass beds (Kollars et al., 2017). Therefore, the conservation dilemma that we describe for the protection of reed beds likely exists for the conservation of multiple types of aquatic plants.

Our findings are in line with studies in other lakes in The Netherlands, the UK and the USA, which have shown that herbivory has a strong inhibiting effect on the colonization and expansion of riparian vegetation in general (Evers et al., 1998; Chaichana et al., 2011; Sarneel et al., 2011, 2014a; Veen et al., 2013; Law et al., 2014; Vermaat et al., 2016). In these studies, Coypu (*Myocastor coypus* Molina), Eurasian beaver (*Castor fiber* L.) and invasive muskrats were identified as the main herbivores, possibly in combination with waterfowl. Generally, it is very difficult to pinpoint which herbivore causes the observed effects. In our study, we are confident that the majority of effects are caused by Greylag geese. We come to this conclusion due to a combination of observations. The grazing traces that we observed on the stems and leaves of reed outside our exclosures did not match those observed by Vermaat et al. (2016, p. 4 of their publication) for muskrat. Furthermore, we regularly observed large amounts of Greylag geese in the reed belt, particularly during molt. We also observed actual grazing by those geese on the reed stems and leaves. Finally, active chasing of geese resulted in a strong increase in reed stem height above the water surface and only few grazing traces on stems were observed in late July, when the reed measurements were done. The grazed stems that we did observe were likely due to cessation of the chasing management by mid-June, and a subsequent increase in goose numbers over the summer. The very low proportion of grazed stems in the year of goose chasing was not significantly different from the proportion of grazed stems in the year before (2012), without goose chasing, however, it should be noted that the proportion of grazed stems was rather low in 2012 (< 0.25), compared to the years 2006–2010 (0.5–1.0).

It is very important to pinpoint which herbivore species causes the lack of reed expansion as this will largely determine the management options. Greylag geese, together with other waterbirds, provide useful ecosystem services (Green and Elmberg, 2014), but also disservices (Buij et al., 2017). The destruction of a reed belt in the water with emergent stems for Great reed warblers and Purple herons to breed in, is an ecological disservice in this respect. The morphology of the grazed reed stand was entirely different from the ungrazed stand. With an average height of about 20 cm above the water level and a low stem density, the grazed reed stand was entirely unsuitable for marsh birds to breed in. Indeed, over the course of the study from 2006 to 2013, the Great reed warbler disappeared from the study area as a breeding bird, as well as the neighboring Purple heron colony in adjacent Lake Loenderveen-Oost, which is under similar grazing pressure (see Sarneel et al., 2014a). Therefore, managers face a dilemma, should they manage one native breeding marsh bird, the Greylag goose, to allow existence of other native marsh species? Apart from birds, other species may profit also from goose removal, as we observed in our exclosures on Lake Terra Nova, where other wetland plants started to grow so well inside the exclosures that they even inhibited growth of the planted reed, due to light competition (Supplementary Figure 1D).

### Management options

Potential successful management to mitigate grazing impact by Greylag geese on reed vegetation could aim at two major aspects: management of the goose population or stimulation of the reed vegetation to make it more robust under grazing. Direct goose population management could be achieved by reducing the amount of geese by preventing them to breed, hunting or chasing the geese away (Eythórsson et al., 2017; Table 2). This would fit the idea of trophic downgrading (Estes et al., 2011): the geese largely lack natural predators, resulting in strong population increases and overgrazing of the environment. However, reducing the population by killing individuals or shaking eggs has been shown to have only limited impact on goose numbers (Klok et al., 2010). In our study, we saw that chasing was successful, as stem density and stem height above the water increased within months, hence the reed belt regained its structure. This measure is labor and time consuming, and it is questionable whether it is successful in the long run, or that chasing needs to be increasingly aggressive to chase birds away. However, concepts of hunting for fear can be useful in this context. Here, the effects of hunting on grazing pressure are much larger than the actual amount of animals killed (Cromsigt et al., 2013). By hunting for fear it may be possible to spatially direct grazing pressure as exerted by natural predators (e.g., Kuijper et al., 2013), in this case away from the reed belts. Furthermore, concepts of scaring geese used for agricultural crop protection may be useful (Simonsen et al., 2017). In our study, we show that practically only the continuous and complete exclusion of geese allowed for reed expansion and establishment, whereas temporal protection was not successful. The root cause of the problem is therefore also the lack of robustness of the reed belt; why can reed not stand grazing?

**Table 2:** Measures to preserve and restore reed beds in the presence of geese through management of geese and increasing the robustness of the reed beds.

**Actions**	**Mechanism**	**References**	**Potential limitations**
Goose population management	Shaking eggs or killing adult geese	Klok et al., 2010; Eythórsson et al., 2017	Keeps population in the growing phase; molting geese from elsewhere may come in
Hunting for fear	Chasing away geese	Cromsigt et al., 2013; Simonsen et al., 2017	Geese may learn that they do not get killed and the fear effect diminishes
Landscape configuration: reducing fragmentation, mosaic and large edge:surface ratio's	Large surfaces of reed beds prevents overgrazing as the agricultural meadows are further away; geese impacts in large reed beds can be positive for other marsh birds	Loonen et al., 1991; Van Eerden, 1997; Vulink and Van Eerden, 1998; Van den Wyngaert et al., 2003	It would not work when large flocks of geese would visit the site just to molt
Cessation of fertilization: do not feed the geese	Making surrounding meadows less attractive for geese: decreasing spill-over grazing on reed	Van Eerden et al., 2005	It may take time for food quality to drop if large amounts of nutrients are still in the soil
Cessation of mowing or grazing	Making the vegetation in the surrounding meadows less attractive, decreasing spill-over grazing on reed	Vulink et al., 2010	Other uses of the meadows may prevent this measure
Water level management	Improving growth and germination conditions of reed by water drawdown in summer making the marsh more resilient to grazing damage	Vulink and Van Eerden, 1998; Coops et al., 2004; Veen et al., 2013; Sarneel et al., 2014b; Van Leeuwen et al., 2014	Geese may eat the newly germinated seedlings as soon as the water level goes up again
Water level management	By water drawdown up to dry fall, geese will not visit the site, preventing herbivory	Amat, 1995; Esselink et al., 1997	Other uses of the water may prevent this measure
Large-scale fencing	Preventing herbivory by fencing off the shoreline for geese, stimulating rejuvenation.	This study	After fence removal geese may still eat the vegetation, herbivory may only be delayed.

In our study site, reed grows typically in belts along the lake shore, a situation which is common for reed (e.g., Liira et al., 2010; Vermaat et al., 2016). As a result, the reed stand has a very high edge:surface ratio. This makes the vegetation vulnerable to grazing. If reed expansion has to be clonally, as is the case in our study area, then grazing on the edges of the vegetation inhibits all the expansion (e.g., Silliman et al., 2013). Geese preferably graze on the edge of a vegetation stand (Bakker et al., 1999; Sarneel et al., 2014a), thus causing an enormous impact, while the reed has little robustness to deal with this disturbance. A very similar situation occurs for other clonal plants, such as Backthorn shrub (*Prunus spinosa* L.), which clonally expands into grassland. Here, clonal expansion can be completely inhibited due to grazing by European rabbits (*Oryctolagus cuniculus* L.) (Smit et al., 2010). In contrast, in the Oostvaardersplassen, which had large surfaces of reed marsh, grazing by molting Greylag geese had positive effects, as the geese created small openings in the dense surface of continuous reed marshes (Van den Wyngaert et al., 2003), which allowed herons and other water birds more easy access to food and predator escape (Vulink and Van Eerden, 1998) and the reed to rejuvenate, inhibiting vegetation succession (Van Eerden, 1997). In contrast, the small, narrow reed zone as found at present in many nature reserves is hypersensitive to grazing. Therefore, we hypothesize that the lack of robustness of the current reed belts can strongly contribute to the problem. These reed belts are so narrow, because expansion is hampered by a range of adverse abiotic conditions over long periods, including eutrophication, accumulation of toxic compounds and wave action (van der Putten, 1997; Lamers et al., 2015). Now that the abiotic conditions are being restored in many lakes (Baastrup-Spohr et al., 2017), the biotic interactions may more effectively hamper the expansion of reed than they would in a healthy, robust system that is not still recovering from abiotic stressors.

Another way of increasing the robustness of the reed bed is to decrease its dependency on clonal expansion. By creating possibilities for reed to recruit via germination, it has an alternative way to expand (Table 2). In particular, the lack of water level fluctuations may form a continuous bottleneck to rejuvenate the reed stands (Coops et al., 2004; Ter Heerdt et al., 2017). Reed cannot germinate under water; hence, dry fall of parts of the shoreline is needed for its germination (Sarneel et al., 2014b; Van Leeuwen et al., 2014). This can be realized by water management which allows water level fluctuations. Indeed, large-scale regeneration of reed beds is possible in this way, but also here, the emerging new reed beds should be at least temporarily free from intensive goose grazing (Coops et al., 2004).

Another very important factor is the spatial distribution of the geese, which concentrates local grazing pressure. The reed zones in many areas are found in a landscape of lakes or ponds in a mosaic configuration with (often fertilized or mown) meadows. Geese are attracted to meadows, even more so when mown and fertilized (Van Eerden et al., 2005). They graze in the meadows and at night, or during molt, rest on the water where they graze on reed (Fox and Kahlert, 2000; Kleyheeg et al., 2017). Hence, a patchwork landscape of meadows and water bodies with reed stands creates a condition of overgrazing: goose populations grow on the fertilized meadows and do not depend on the reed for survival, so they can afford to overgraze the reed belt and remove it completely, without population consequences. This typical condition of external subsidies leading to overgrazing is well-known for Lesser snow geese, where population growth subsidized by farm fields on the winter grounds, causes destruction of the arctic tundra where they breed, due to overgrazing (Jefferies et al., 2004). Here, we show that this overgrazing is not unique to the Arctic, but can occur equally well with geese in temperate regions overgrazing nature reserves, when being subsidized by fertilized agricultural meadows. A solution to this problem should be found at the landscape scale. By avoiding an intense mixture of short distance combinations of meadows and ponds, the grazing pressure on reed is expected to be lower. Additionally, meadows surrounding reed beds should not be fertilized. Lastly, minimalized mowing management may be a way to indirectly protect reed from grazing, as many geese are sensitive to facilitation, meaning that they are attracted to short lawn vegetation (Vulink et al., 2010). For instance, city parks, golf courses and roundabouts along highways are attractive sites for geese for this reason, combined with a lack of predation. As a result of collateral damage, no helophyte vegetation will emerge in ponds in this type of habitat.

## Conclusions

We demonstrated that removal of Greylag geese by either fencing or management created tall, dense reed belts growing in the water along the lake shore, whereas these were largely absent in the presence of geese. Overall, we observed a strong grazing effect on reed expansion of both existing reed stands and establishing reed plants. Thus, there is a conservation dilemma how to preserve and restore reed belts in the presence of high densities of Greylag geese. We propose that the effects of the geese can be mediated by goose population management, but also by increasing the robustness of the reed marshes, by making them larger and reducing the edge:surface ratio, allowing water level management both to stimulate reed growth and germination and also to prevent geese access to the reed. Furthermore, at the landscape level, spatial configuration with larger wetland reserves surrounded by unmown, unfertilized meadows will reduce geese grazing pressure on reed.

## Author contributions

EB, JS, and GT initiated and designed the work, EB, JS, and NH collected the data, CV, EB, and JS analyzed the data, EB, JS, and CV wrote the paper, all authors contributed to data interpretation and critically commented on the draft manuscript.

### Conflict of interest statement

The authors declare that the research was conducted in the absence of any commercial or financial relationships that could be construed as a potential conflict of interest.

## Supplementary material

Supplementary Figure 1 Design and development of the reed planting experiment at Lake Terra Nova. **(A)** Wooden bank protection to create a sheltered habitat for reed development. **(B)** Freshly planted reed plants. **(C)** Exclosure at the start of the experiment, planted reed is visible inside and outside (on the right side of) the exclosure. **(D)** After one year, the planted reed grew very tall inside the exclosures, whereas also other helophytes grew well inside the exclosures. Pictures by the authors.

Supplementary Figure 2 **(A)** Characteristic reed belt growing at the fringe of the shore of Lake Waterleidingplas. Part of it grows on the shore and part of it extends into the water, up to about 2 m from the shore. The bright green patch of reed is growing in one of the exclosures in the first year on 28 June 2006. Several more exclosures are recognizable in the background, to the right of the first exclosure, with the green patches of reed. The exclosures extend 6 m into the open water. **(B)** Reed stems and leaves grazed by Greylag geese. Both the leaves and the living stems, the green stem on the right of the picture, and the shorter greenish bitten off stem to the left of it both show the irregular fringe left at leaf and stem after goose grazing. The shorter grayish stem immediately to the left of the green stem with leaves is a stem from last year and dead.
