# Supplementary figures and images for: High Grazing Pressure of Geese Threatens Conservation and Restoration of Reed Belts
Source: Front Plant Sci. 2018 Nov 12;9:1649. doi: 10.3389/fpls.2018.01649 (PMC6240796; doi:10.3389/fpls.2018.01649)

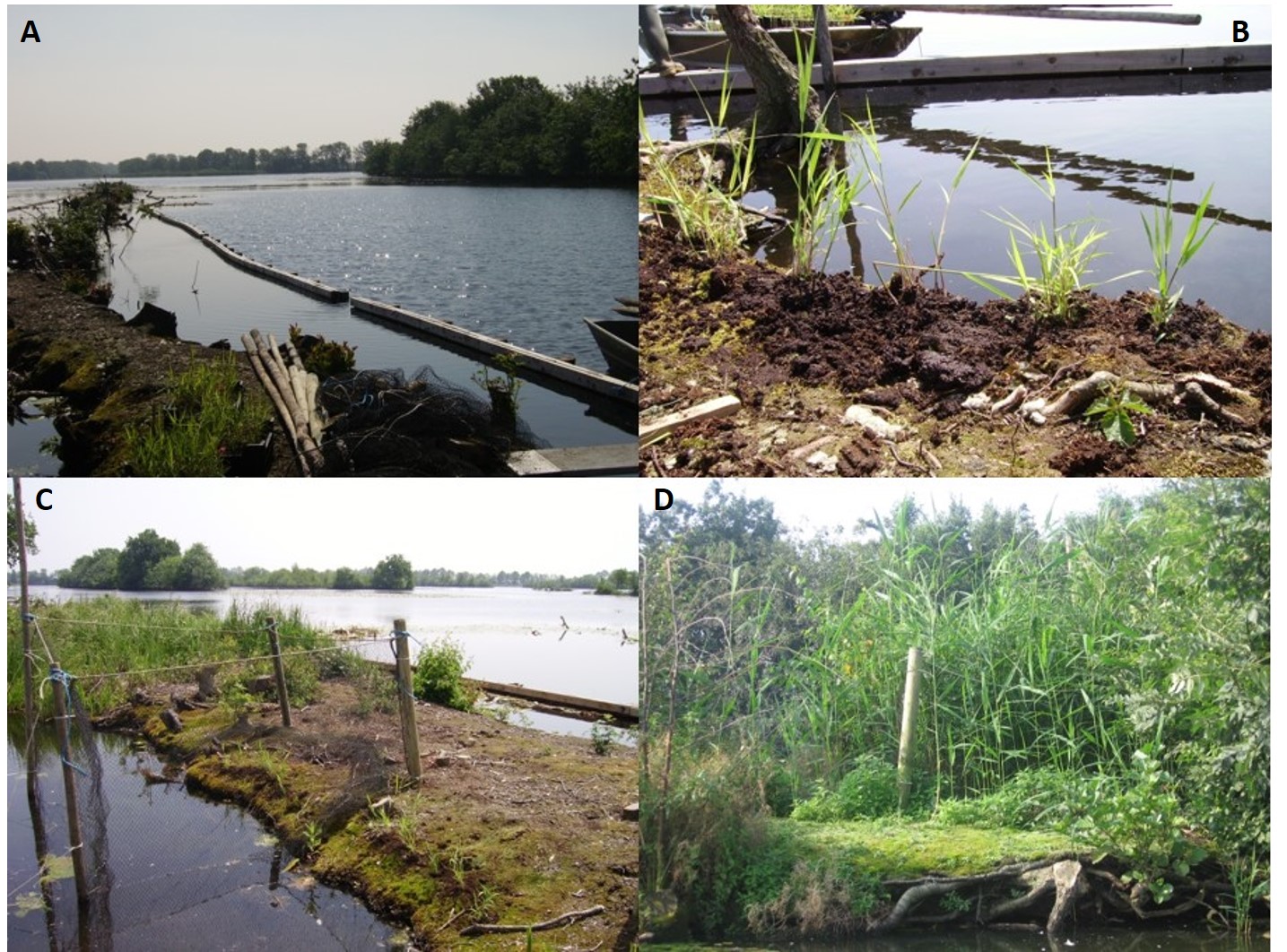

Supplement: Supplementary Figure 1 — Design and development of the reed planting experiment at Lake Terra Nova. (A) Wooden bank protection to create a sheltered habitat for reed development. (B) Freshly planted reed plants. (C) Exclosure at the start of the experiment, planted reed is visible inside and outside (on the right side of) the exclosure. (D) After one year, the planted reed grew very tall inside the exclosures, whereas also other helophytes grew well inside the exclosures. Pictures by the authors. [file Image_1.jpg]

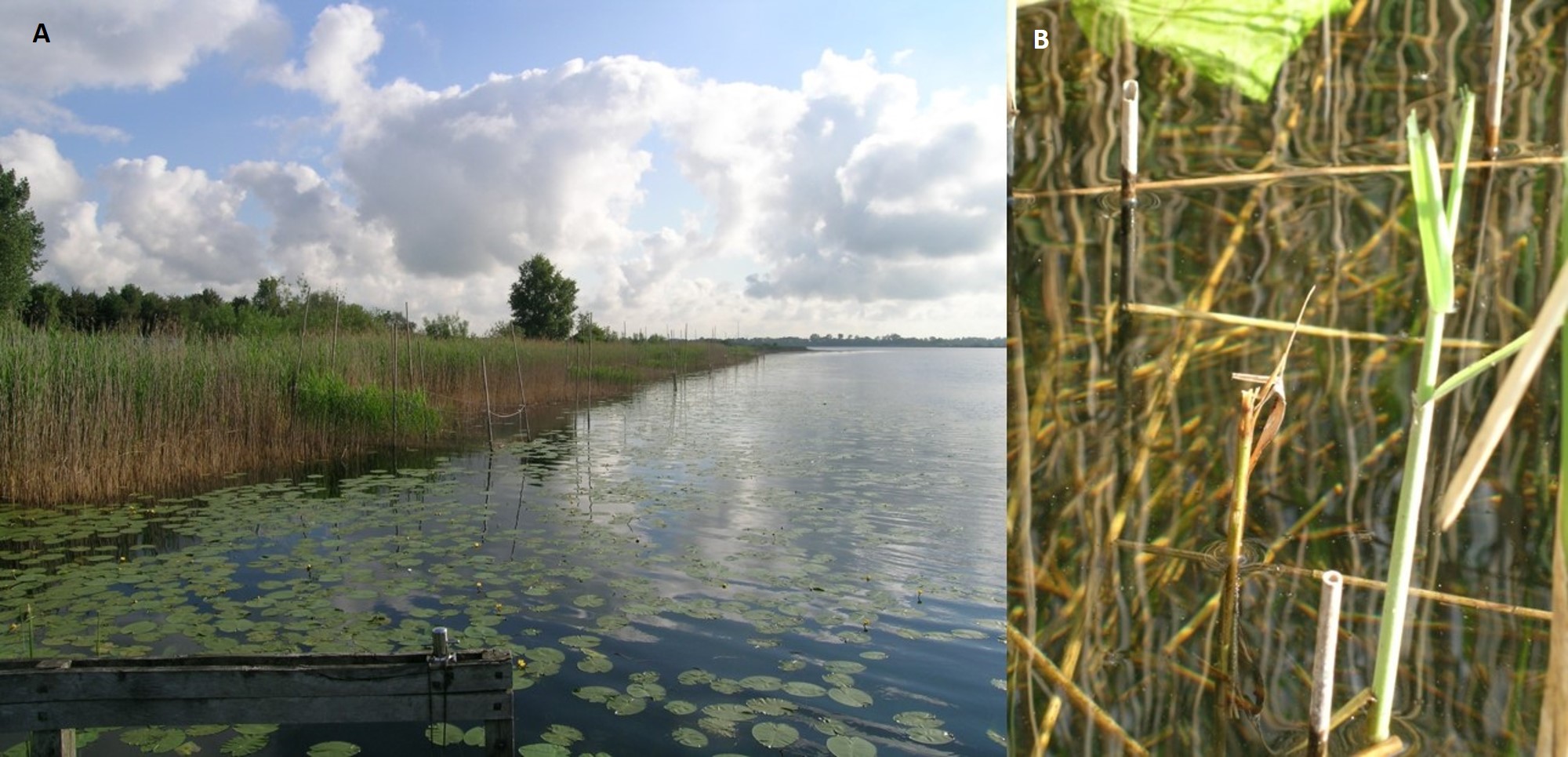

Supplement: Supplementary Figure 2 — (A) Characteristic reed belt growing at the fringe of the shore of Lake Waterleidingplas. Part of it grows on the shore and part of it extends into the water, up to about 2 m from the shore. The bright green patch of reed is growing in one of the exclosures in the first year on 28 June 2006. Several more exclosures are recognizable in the background, to the right of the first exclosure, with the green patches of reed. The exclosures extend 6 m into the open water. (B) Reed stems and leaves grazed by Greylag geese. Both the leaves and the living stems, the green stem on the right of the picture, and the shorter greenish bitten off stem to the left of it both show the irregular fringe left at leaf and stem after goose grazing. The shorter grayish stem immediately to the left of the green stem with leaves is a stem from last year and dead. [file Image_2.jpg]
